# Acute Aortic Syndromes from Diagnosis to Treatment—A Comprehensive Review

**DOI:** 10.3390/jcm13051231

**Published:** 2024-02-21

**Authors:** Cosmin M. Banceu, Diana M. Banceu, David S. Kauvar, Adrian Popentiu, Vladimir Voth, Markus Liebrich, Marius Halic Neamtu, Marvin Oprean, Daiana Cristutiu, Marius Harpa, Klara Brinzaniuc, Horatiu Suciu

**Affiliations:** 1 I.O.S.U.D., George Emil Palade University of Medicine, Pharmacy, Science, and Technology of Targu Mures, 540139 Targu Mures, Romania; cosmin.banceu@umfst.ro (C.M.B.);; 2 Department of Surgery M3, George Emil Palade University of Medicine, Pharmacy, Science, and Technology of Targu Mures, 540139 Targu Mures, Romania; 3 Emergency Institute for Cardiovascular Diseases and Transplantation Targu Mures, 540136 Targu Mures, Romania; 4 Department of Surgery, Division of Vascular Surgery, Stanford University School of Medicine, Palo Alto, CA 94305, USA; 5 Faculty of Medicine, University Lucian Blaga Sibiu, 550169 Sibiu, Romania; 6 Sana Cardiac Surgery, 70174 Stuttgart, Germany; 7 Swiss Federal Institute of Forest, Snow and Landscape Research WSL, 8903 Birmensdorf, Switzerland; 8 Institute of Environmental Engineering, ETH Zurich, 8039 Zurich, Switzerland; 9 Mathematics and Statistics Department, Amherst College, Amherst, MA 01002, USA; 10 Department of Anatomy, George Emil Palade University of Medicine, Pharmacy, Science, and Technology of Targu Mures, 540142 Targu Mures, Romania

**Keywords:** acute aortic syndrome, acute aortic dissection, intramural hematoma, penetrating atherosclerotic ulcer, traumatic aortic injury, aortic centres, Stanford A, Stanford B, Stanford non-A non-B, aortic surgery

## Abstract

This work aims to provide a comprehensive description of the characteristics of a group of acute aortic diseases that are all potentially life-threatening and are collectively referred to as acute aortic syndromes (AASs). There have been recent developments in the care and diagnostic plan for AAS. A substantial clinical index of suspicion is required to identify AASs before irreversible fatal consequences arise because of their indefinite symptoms and physical indicators. A methodical approach to the diagnosis of AAS is addressed. Timely and suitable therapy should be started immediately after diagnosis. Improving clinical outcomes requires centralising patients with AAS in high-volume centres with high-volume surgeons. Consequently, the management of these patients benefits from the increased use of aortic centres, multidisciplinary teams and an “aorta code”. Each acute aortic entity requires a different patient treatment strategy; these are outlined below. Finally, numerous preventive strategies for AAS are discussed. The keys to good results are early diagnosis, understanding the natural history of these disorders and, where necessary, prompt surgical intervention. It is important to keep in mind that chest pain does not necessarily correspond with coronary heart disease and to be alert to the possible existence of aortic diseases because once antiplatelet drugs are administered, a blocked coagulation system can complicate aortic surgery and affect prognosis. The management of AAS in “aortic centres” improves long-term outcomes and decreases mortality rates.

## 1. Introduction

**Figure 1:**
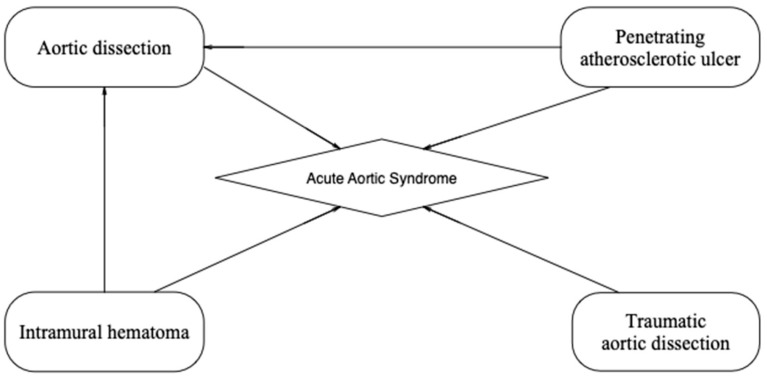
Acute Aortic Syndrome Spectrum.

Disorders of the thoracic and abdominal aorta known as acute aortic syndromes (AASs) are potentially life-threatening, typically symptomatic and necessitate prompt surgical assessment. The term AAS refers to a diverse group of disorders that share a common set of signs and symptoms, the most prominent of which is aortic discomfort. It was first used in 1998 [1] and thoroughly defined in 2001 [2]. Aortic dissection (AD), intramural hematoma (IMH), penetrating atherosclerotic ulcer (PAU) and traumatic AD (TAD) are the four acute aortic diseases considered as AAS, see Figure 1.

Knowledge of the pathologic spectrum of AAS helps to clarify the origin and course of aortic lesions [3,4]. AAS can be categorised into two groups based on whether the ascending aorta is involved (Stanford type A) or not (Stanford type B). A unique disease known as non-A non-B AAS occurs when a lesion is limited to the aortic arch only or when a lesion that originated distal to the left subclavian artery retrogradely spreads to the arch instead of accessing the ascending aorta [5]. Any AD with an entrance tear that starts distal to the innominate artery ostium is classified as a type B AAS [6].

Acute AD is a highly prevalent and potentially deadly condition, necessitating prompt diagnosis and treatment. If not treated, mortality can be up to 90% after three months, and acute mortality can be 1–2% per hour after the onset of symptoms [7,8]. A tear in the aortic intima causes AD, which is characterised by a blood column entering the medial layer of the aorta and resulting in a “hydraulic endarterectomy” [9]. This creates a septum made of medial and intimal aortic tissue that separates the aorta’s real and false lumens. In many people, the aorta’s detached outer layer, which consists of adventitia and aortic medial tissue, is robust enough to prevent the aorta from rupturing into the pericardial cavity and causing fatal cardiac tamponade, or into the pleural cavities and causing deadly exsanguination. In these cases, aortic branch occlusion is determined by the size of the dissecting hematoma [10]. This hematoma can induce blockage of vital aortic branches, such as those supplying the brain, kidneys, abdominal viscera and limbs. The dissection flap arises from a tear in the proximal aorta and spreads distally for a varied length. Paraplegia can occur when the lumbar and intercostal arteries are occluded. When the dissecting hematoma grows near the aortic root, it may obstruct the coronary artery ostia, which could cause myocardial infarction or damage the aortic valve, which may then cause aortic regurgitation.

A highly prevalent trigger for acute AD is hypertension [7]. Approximately 20% of individuals have severe forms of cystic medial degeneration. Aortic coarctation, bicuspid valve, dilatation of the ascending aorta and aortic root, collagen vascular disorders, extreme isometric exertion, pregnancy and aortic inflammatory diseases are additional risk factors. Acute AD is most frequently characterised by abrupt, intense chest discomfort [7]. Acute AD may occur in the absence of pain, and additional variations of back and chest discomfort have been observed. The anterior chest is typically where type A dissection pain is felt, though it can also radiate to the back, neck or belly. The pain associated with type B dissection typically manifests as both posterior chest and abdominal pain. Additional symptoms include a coma induced by obstruction or serious blockage of the brachiocephalic arteries and syncope, which is caused by hypotension linked to cardiac tamponade or aortic rupture. Hypoperfusion of the renal and mesenteric arteries causes abdominal pain. In the context of acute AD, mesenteric ischemia can result in a death rate of up to 87% and renal failure in the range of 50–70% [7]. Obstruction of the infrarenal aorta and iliofemoral arteries causes acute limb pain and pulselessness.

## 2. Clinical Presentation and Diagnosis

### 2.1. Challenges of Diagnosis

To establish a correct diagnosis of AAS, an in-depth examination must be performed, which involves investigations allowing for the exclusion of several other conditions that are part of the differential diagnosis. The diagnosis of AAS is difficult due to three key factors: a modest prevalence, a lack of precise biomarkers and a highly varied clinical course [11,12]. Thus, the key to diagnosing AAS is to retain an elevated level of medical suspicion. Several initiatives—including AAS training sessions, educational materials, computerised diagnostic instruments and algorithms created especially for patients with chest pain—may increase the level of awareness [13,14,15]. A three-step process has been designed to make emergency room diagnosis easier.

#### 2.1.1. Phase 1

The first phase of this process entails determining the likelihood of AAS using three categories: risk factors, physical examination results and characteristics of pain. Risk factors for AAS are widely recognised. The most common, severe chronic hypertension, is closely linked to the emergence of AAS. Indeed, individuals with hypertension have a risk of AAS more than twice as high as those without it, and 54% of the population has a risk of AAS due to hypertension [16]. Other risk factors that should be considered include bicuspid aortic valve, aortic valve disease, previous family history of aortic disease, Marfan syndrome and Loeys–Dietz syndrome. In addition to risk factors, a highly prevalent clinical complaint among patients with AAS is chest discomfort [13,17,18,19]. According to descriptions, the “aortic pain” is sudden, sharp, strong and tearing, and often radiates towards the destination of the lesion’s advancement in accordance with the implicated aortic branches. Patients with type A AAS most often have pain in the chest, whereas patients with type B AAS typically experience aortic pain in the back. Experiencing AAS without discomfort is less common. Finally, a thorough physical examination may show common indications of AAS, such as an aortic regurgitation murmur or pulse deficit/asymmetric pulses. Acute aortic syndrome should be discussed in patients hospitalised for severe hypotension, syncope or shock, especially in those who additionally presented chest pain. This preliminary likelihood evaluation is advised for class I [13,15]. If more than one risk factor is found, this would prompt concerns about possible AAS and direct further action.

#### 2.1.2. Phase 2

The second phase entails the standard diagnostic procedures that are required for any patient experiencing chest pain, including an electrocardiogram (ECG), a chest X-ray and laboratory testing for biomarkers [14,20]. This is a crucial step in the diagnostic procedure that allows differentiation between the two most common serious illnesses that cause chest pain: pulmonary embolism and acute coronary syndrome (ACS). ACS can be diagnosed with great sensitivity when troponin levels and ECG are combined [20]. Chest pain originating from the heart, such as in myocardial infarction, can be distinguished with an ECG. AAS and myocardial infarction can both manifest simultaneously, although cases like this require more research and cautious handling. Because antiplatelet medications required for ACS may complicate therapy and significantly decrease the prognosis of patients with AAS, differential diagnosis between AAS and ACS is crucial. However, a high troponin level does not mean that AAS is not present. Elevated troponin levels in patients with AAS can be attributable to myocardial ischemia worsened by acute aortic regurgitation or hypotension, or they can result in death if the dissection flap involves a coronary artery. The use of D-dimer values for AD diagnosis has been extensively demonstrated in numerous investigations [21,22,23]. D-dimer values boost the effectiveness of the diagnostic approach when they are combined with an evaluation of the likelihood of developing AAS [22,24]. As a result, the sensitivity and specificity are 98.8% and 57.3%, respectively, whenever a patient exhibits one or more risk factors for AAS and the D-dimer is negative. The negative likelihood ratio for developing AAS therefore amounts to nearly 100% [22].

#### 2.1.3. Phase 3

The second stage is crucial because, in patients presenting with “aortic pain”, elevated D-dimer values, normal troponins and a normal ECG, all suggest the possibility of AAS/pulmonary embolism, which necessitates imaging tests, i.e., the third stage, which will yield a conclusive diagnosis [15,25]. The core of the diagnostic process is imaging, which involves the application of magnetic resonance imaging (MRI), computed tomography (CT), ultrasound (US) or chest radiography. Basic chest radiography may detect AAS with up to 64% sensitivity and 86% specificity. The characteristics observed on a chest X-ray include tracheal shift, aortic kinking, widening of the mediastinum, widening of the aortic notch and a twofold increase in the frequency of the aortic shadow [26]. In the emergency room, CT is the favoured imaging diagnostic for confirming the development of AAS. This method is ideal because of several benefits: quick collection times, widespread accessibility in emergency rooms in most hospitals and comprehensive anatomic evaluation of the overall aorta and aortic branch vessels [25,27]. A CT scan can recognise AAS with a normative sensitivity of up to 95%. Observed values have ranged from 87–100% [28,29]. All patients with intermediate/high diagnosis of AAS should have a full aortic CT scan, regardless of the contrast [30].

### 2.2. Assessment Methods

Both transthoracic echocardiography (TTE) and transesophageal echocardiography (TEE) can aid in the diagnosis of AAS, but their efficacy differs greatly. TTE is useful for diagnosing both proximal dissection and its consequences in an acute scenario. However, there are numerous other locations throughout the entire aorta where views may be restricted. Focus TTE may also help to evaluate other pertinent conditions, such cardiac function, aortic valve regurgitation and pericardial effusion [13,31]. However, TEE enables the US tool to approach the aorta extremely closely, resulting in 99% sensitivity and 89% specificity in one study [32]. In an emergency situation, TEE becomes much less accessible and more operator-dependent. Additionally, an examination that necessitates esophageal intubation is significantly more intrusive. A bedside TTE and CT scan combination is a great diagnostic strategy, since it enables the assessment of likely problems related to AAS and a view of the entire length of the aorta.

Because there are few MRIs available for use during an emergency and because AASs are serious, MRIs are rarely employed as the main method of acute investigation. Whenever initial investigations are not definitive, this modality—which is a highly sensitive and specific means of identifying all forms of AAS—can be employed. TEE has been implemented during surgery to provide necessary information, and to promptly assess outcomes during open surgery and endovascular therapy [33].

In patients with AAS, the use of chest X-rays and ECGs is restricted to eliminating other conditions that manifest as chest discomfort. Chest X-rays for type A lesions can show enlarged mediastinum, although this result is absent in 20–28% of dissections [34,35]. AD cannot be diagnosed only by X-ray. Heart troponin T levels are often higher in patients with AAS and are linked to a later diagnosis [36]. TEE and CT or MRI are part of the first diagnostic assessment [37]. According to published research, TEE has greater specificity and sensitivity (86–100% and 90–100%, respectively) for acute dissection than CT (95–100% and 94–98%, respectively) [28,38,39,40,41,42]. TTE, in contrast, performs less well, with a median sensitivity of 86.9–100.0% and a specificity of 81.2–91.0% (81.1%) [43,44].

Acute dissection must be diagnosed as soon as possible. When imaging is not an option (due to a lack of scanners or a patient’s declining clinical condition, for example) serologic biomarkers that indicate nascent aortic wall injury are a desirable diagnostic tool. D-dimer is now the best researched among those biomarkers [21] and has a minimal threshold level of 0.5 μg/mL, a sensitivity of 51.7–100.0% (median, 93.5%) and a specificity of 32.8–89.2% (median, 54.0%) [21,45,46,47,48]. Moreover, higher in-hospital mortality has been linked to higher D-dimer levels [49]. The following biomarkers were also studied: matrix metalloproteinase [50], soluble lectin-like oxidised low-density lipoprotein receptor [51], smooth muscle myosin heavy chain [52] and soluble elastin fragments [53]. Nevertheless, the dearth of randomised controlled trials precludes any inferences about their capacity to boost results.

Between 35 and 37% of the ECGs in the International Registry of Aortic Dissection (IRAD) and another cohort were normal. Some studies have shown that 50–70% of patients had enlarged mediastina on their chest X-rays [34,54]; of those, 26% had pleural effusion [54]. The sensitivity and specificity of TEE were 96.5–99.6% and 92.3–98.5%, respectively [55,56,57]. After receiving medical attention for a mean of 450 days, 44 patients with a simple type B IMH were monitored; the disease advanced in 87% of the individuals exhibiting an early intimal abnormality, whereas this occurred in only 9% of individuals lacking such an abnormality (*p* < 0.001). One study found that 80% and 40% of patients with IMH with focal dissection showed five and eight years, respectively, without death linked to dissection (Table 1) [58].

CT and MRI are the gold standards for the diagnosis of IMH. Furthermore, CT identification of intimal defects (erosion of the vessel wall in discrete locations) in patients with IMH is associated with progression to dissection [59].

## 3. Treatment of Patients with AAS

The two types of AAS are those that impact the ascending and descending aortas. Surgical intervention is the final therapy for situations affecting the ascending aorta. Immediate surgical intervention is required only for rapidly progressing static AAS impacting the descending aorta and causing organ or limb malperfusion, unbearable pain or danger of rupture. Static AASs affecting the descending aorta are typically managed conservatively [60].

To halt the advancement of the dissection or prevent aortic rupture, early therapy for patients with AAS in emergencies concentrates on lowering systolic blood pressure below 120 mmHg and lowering the rate of change of blood pressure (dP/dt). An injectable beta-blocker, like labetalol, is the cornerstone of medical therapy for keeping the heart rate around 60, which is called “impulse control”. Non-dihydropyridine calcium channel blockers could be an option for patients who cannot tolerate beta-blockers. Vasodilators may be administered in addition to these.

In the examined trials, the observed 30-day or in-hospital mortality for type A acute AD ranged from 13–17% for open surgical treatment and from 0–16% for thoracic endovascular aneurysm repair (TEVAR). For people with type A AAS, urgent open surgical correction is the recommended course of action. The primary goals are to prevent aortic rupture, fix aortic regurgitation and reroute blood flow to the correct lumen [14]. A supracoronary tube graft, with or without valve replacement or repair, may be adequate to accomplish this purpose provided the aortic root is unaffected. The standard procedure for treating aortic root inclusion is a Bentall–De Bono operation, which can be combined with a mechanical or biological valve replacement [61,62].

Nevertheless, high-volume centres and skilled surgeons must consider various other approaches. In certain patients, valve-sparing aortic root surgeries, including the David or Yacoub approaches, can be explored [62,63]. Aortic root substitution using a composite valve tube or aortic valve resuspension is necessary for people with aortic annulus growth or entry tears at the aortic root and for the majority of people with connective tissue illnesses.

An open method has historically been used to treat ascending AD. The goal of treatment is to close or excise the most proximal intimal tear and all successive tears in order to eradicate the false lumen. As an alternative, artificial grafts may be used to strengthen the aortic wall. Aortic valve inadequacy and coronary artery injury can result from proximal extension into the aortic valve. These can be fixed either by replacing the complete valve or by resuspending the valve.

In patients with type A AAS, “hemiarch” replacement of the aortic arch with no involvement of the supra-aortic trunks remains the conventional surgical technique. The safety and efficacy of total arch replacement are becoming more and more clear [64,65,66]. Therefore, in centres with appropriate expertise, a total arch replacement procedure must be considered in the event of any of the following conditions: a dilated aortic arch, an entry tear at the aortic arch, arch or proximal descending re-entrance tears or a dilated proximal descending aorta [14,66,67]. This strategy enables distal aortic remodelling and shields against additional operations. Employing a hybrid prosthesis, the “frozen elephant trunk” method proved to be a workable solution in AAS scenarios, resulting in positive outcomes. By joining more endoprostheses to the distal portion of the hybrid prosthesis, it also becomes feasible to implement an endovascular restoration of the distal descending and abdominal aorta. The challenge of managing dissection affecting the aortic valve and root is the main drawback of the endovascular method.

In the IRAD, surgical intervention was used to treat 72% of patients with type A acute AD. When a patient was too old, had too many coexisting conditions, refused treatment or passed away before a scheduled surgery, medical intervention was employed. In-hospital mortality following open surgery was 26%, whereas that following medical management was 58% [38]. IRAD data were used to identify four different periods—hyperacute (0–24 h), acute (2–7 days), subacute (8–30 days) and chronic (≥30 days)—for the onset of symptoms and emergency department attendance. Nevertheless, the observational study design limits these findings [68]. Twenty to thirty percent of 2317 individuals with acute type A dissection in a large German registry had neurological impairment at presentation; 12.3% of these patients recovered after surgery and 9.5% of these individuals had additional neurological problems after surgery. A fresh episode of postoperative neurological impairment was associated with longer operating time, dissection of the supra-aortic arteries and malperfusion syndrome [69]. Between 2006 and 2015, nine off-label and five on-label procedures were conducted in the sole US Food and Drug Administration-approved, physician-sponsored investigational device exemption study of endovascular therapy for type A AD [70]. Acute and chronic type A ADs affected six patients and five patients, respectively. All surgeries were practically effective, with a 7.1% 30-day mortality rate.

For type B AAS, problems such as hemodynamic instability, malperfusion, rapid aortic dilation and aortic rupture should be addressed by TEVAR. Additional dangerous conditions that must be considered include refractory discomfort or hypertension, a large proximal entrance tear, an aortic diameter greater than 44 mm and a false lumen diameter greater than 22 mm [6,13]. Since TEVAR has demonstrated positive outcomes and few periprocedural problems, it can be postponed whenever feasible [71,72]. All other patients with type B AAS should be treated.

For type B acute AD, 30-day or in-hospital mortality ranged from 0–27%, 13–17% and 0–18% for medicinal treatment, open surgical surgery and TEVAR, respectively. In the IRAD study, in-hospital mortality for type B acute AD managed with medicinal therapy was 9.5%, but it reached 29% for the surgical cohort [73]. Malperfusion syndrome or indications of a periaortic hematoma in the surgical cohort suggested the need for surgery. Therefore, it is possible that the two groups’ different levels of illness severity affected the outcomes.

In a propensity-matched study, Fattori et al. [74] compared 276 patients receiving TEVAR to 853 patients receiving medical treatment for type B dissection. Although TEVAR patients had more problems upon admission, hospital mortality did not differ between the two groups, and TEVAR patients had a decreased five-year cumulative risk of death (15.5% vs. 29.0%; *p* = 0.02) [74]. An experimental device exemption study using TEVAR for complex type B dissections reported a 30-day mortality rate of 8% (Table 2) [75].

In patients with uncomplicated acute type B AD, the Level IB Acute Dissection: Stent Graft or Best Medical Treatment (ADSORB) trial compared medical therapy with TEVAR in a randomised controlled study. “Favourable aortic remodelling” at one year was the main goal. There were no aortic ruptures in either group and the amount of aortic dilatation was comparable in the two groups. In contrast to patients receiving TEVAR, participants receiving medical therapy had a lower rate of false lumen thrombosis (97% vs. 43%; *p* < 0.001). Additionally, compared to the medically treated group, the TEVAR group showed better aortic remodelling at the one-year follow-up [76]. Despite addressing chronic type B dissections, the Investigative Study of Stent Grafts in Aortic Dissection with Extended Length of Follow-up (INSTEAD-XL) trial offers some of the finest Level IB evidence for long-term results following TEVAR in uncomplicated type B dissection [71]. INSTEAD-XL found that, at the five-year assessment, TEVAR was linked to improved results compared to solely medical treatment for both disease progression (4.1% vs. 28.1%; *p* = 0.004) and endpoint (6.9% vs. 19.3%; *p* = 0.04). However, aorta-specific mortality for TEVAR was higher in the first 12 months (7.5 vs. 3.0). TEVAR failed to lower the all-cause mortality rate (11.1% vs. 19.3%; *p* = 0.13) [71].

Aortic intramural hematoma (AIH) requires particular attention in a few areas. Though less common than ascending AD, AIHs are managed similarly because of the possibilities of progression to dissection, aneurysm formation or aortic rupture. Whenever feasible, endovascular stenting is used for AIH impacting the ascending aorta, while surgery or watch-and-wait therapy is used for instances impacting the descending aorta [77]. In asymptomatic individuals with type A AIH, early medicinal therapy combined with a careful waiting approach may be a safe course of action. In such a scenario, it is necessary to provide serial clinical and radiological assessment and to conduct surgery in the event of hemodynamic deterioration or the emergence of high-risk imaging signals [14,78,79]. Additionally, for people with type B AIH, the standard unidentified variables in TEVAR include the lack of a healthy aortic proximal landing zone and an evident entrance tear that needs to be closed. Patients with type B AIH are often treated medically, and the development of ulcer-like projections warrants close imaging monitoring because they are linked to an increased risk of complications, particularly if they are detected on the first CT scan [80,81,82].

Medicinal therapy was found to be appropriate for patients with “moderate” IMHs in a study involving 86 cases of AAS. Patients with moderate hematomas include patients without hemodynamic instability, persistent pain, impending rupture and ruptured aneurysm. Definitive surgical treatment was recommended for patients with “severe” IMHs who exhibited these symptoms. Of 26 patients who were treated medically, six (23%) experienced spontaneous regression and seven (27%) ultimately underwent surgery [83]. Among 27 patients who had conservative care for a type B IMH and were monitored for an average of 33 months, 47% experienced regression, 14% stayed stable and 39% advanced to AD or enlargement [34]. In a different trial, medical therapy plus TEVAR was compared to early medical therapy for IMHs complicated by intimal erosion. Ninety percent of medically treated patients showed regression at a mean follow-up of 17.6 months, and 45% of patients had full clearance of their IMH. Every patient receiving TEVAR experienced resolution [84].

Song et al. examined 127 individuals with type A and type B IMHs. Patients with type A IMHs had greater mortality rates than type B patients (7% vs. 1%) and more frequent pericardial and pleural effusions [85]. Nevertheless, rates of regression and progression to dissection were comparable. For type A IMH in the IRAD, the in-hospital mortality rates were 24% for surgical therapy and 40% for medical therapy. The in-hospital mortality rates for type B hematoma were 20% for surgical therapy and 4% for medicinal treatment [54]. TEVAR and medical therapy were compared for 56 individuals with type B IMHs. Patients who had sustained chest or back pain despite receiving the most aggressive medical care, had a maximum aortic diameter greater than 45 mm or had hematoma thickness greater than 10 mm were eligible for TEVAR. There was neither mortality nor progression in the TEVAR group, leading to 100% clinical success.

Medical therapy is a safe way to treat isolated asymptomatic PAU, which is frequently incidentally discovered on imaging obtained for other purposes. Aortic rupture, pseudoaneurysm formation and aneurysm formation are more likely to occur in cases of symptomatic PAU. Surgical therapy is often necessary because of the comorbidities and advanced age of patients who are commonly diagnosed with PAU. Endovascular surgery with stent grafts provides the largest reduction in mortality, over non-interventional management [86]. However, because these studies are observational, the quality of evidence is limited.

## 4. Discussion

### 4.1. The Role of Multidisciplinary Teams in Treating AAS

Treatment for acute aortic pathology should be centralised in “aortic centres” due to substantial evidence for a volume–outcome link [87,88,89]. The goals are very clear: lower early mortality, prevent repeat surgeries and improve long-term results.

The “aorta code”, an organised emergency care approach that is always accessible and can be initiated from emergency rooms of low-volume hospitals, should be developed to overcome the obstacles that impede early detection and efficient management of AAS. The aorta code serves three main purposes: (1) In order to diagnose AAS earlier, emergency care providers should be more knowledgeable about it. (2) Patients should be transferred to an aortic centre as soon as possible to shorten the time between diagnosis and treatment. (3) By employing highly skilled aortic surgeons, the best course of action can be provided, thereby improving clinical outcomes.

The objective of this code is to provide patients with AAS with standardised, optimised care using a systematic procedure, from the emergency department to the operating room [90,91]. An “aorta team” is employed for diagnosis, treatment and aftercare [15]. Practitioners from multiple areas of expertise, such as clinical cardiologists, cardiac imaging specialists, cardiac surgeons, vascular surgeons, radiologists, vascular interventional radiologists, anesthesiologists and others, are required to possess a high level of knowledge about the care of patients with AAS. The multidisciplinary group needs to work together well. To determine the optimal course of treatment for each patient—open surgery, hybrid vascular and endovascular operations, a full endovascular approach or conservative management—early consultation with the aortic team is crucial [13,15]. Following initial hospitalisation, every patient should undergo systematic monitoring and surveillance at a clinic experienced in aortic care.

Apart from preventive measures, the best way to enhance clinical results is by centralising treatment of AAS (high-volume surgeons in high-volume centres) [17,92,93,94,95]. It should be made clear, nonetheless, that the experience of an individual surgeon is very important for achieving the best results [93,95,96]. Having low-volume surgeons treat patients with AAS at a high-volume centre is not ideal.

### 4.2. Prevention of AAS

Preventive surgery, medical therapy, controlling hypertension and gathering a thorough family history are all essential preventive procedures that can detect individuals who are at risk for AAS.

It has become widely known that certain aortic problems can be inherited [97,98]. Regardless of the presence of symptoms related to aortic disease, a family history of AD is an important risk factor for AAS. The general likelihood of developing AAS is influenced by both genetic and environmental factors; therefore, it is prudent to perform regular imaging and genetic screening of individuals with a family history of both AAS and aortic aneurysm. Tight supervision of risk factors and close observation of patients with AAS could reduce the incidence of this medical condition.

One established indication of risk of AAS is aortic aneurysm [13]. As a result, suggestions for preventive aortic restoration, with a focus on aneurysm diameter, in such patients are provided by the guidelines of professional associations [13,15,99]. Nevertheless, their effectiveness in lowering the prevalence of AAS in the general population is unknown. The diagnosis of aortic aneurysm has become more common over time, and surgical repair rates have also increased [100,101]. However, interventions for AAS continue to rise concurrently [102,103].

Since most patients with type A acute ADs have ascending aortic diameters less than 55 mm, the guidelines’ suggestions appear to be overly cautious. Based on the available data, the likelihood of having an aortic diameter larger than 55 mm is approximately 20% and, if the diameter is lower, it is less than one in a million [104]. With a diameter less than 3.5 cm, the normal aorta often presents a deceiving appearance. The prevalence of individuals falling within this smaller size range contributes to the aortic size paradox [105]. Setting a limit of 50 mm or below would undoubtedly prevent some AAS, but doing so would bring higher risks of morbidity and death from ascending aortic surgery. Furthermore, after adjusting for body surface area, the Genetically Triggered Thoracic Aortic Aneurysms and Cardiovascular Conditions (GenTAC) registry revealed that women experienced dissection at narrower aortic diameters than men despite having comparable aortic sizes [106]. Women’s body sizes are often smaller than men’s; therefore, it is possible that women are receiving less treatment since present guidelines depend on aortic diameters. Regardless, relying only on the size criterion to decide on when to address an aortic aneurysm is inadequate.

A history of hypertension has been shown to be a reliable indicator of type A acute AD [104]. Controlling blood pressure thus becomes vitally important. Given that half of all people with hypertension globally are ignorant of their blood pressure, it appears that patient and clinician education regarding blood pressure is required [107]. Given that AASs are uncommon but many individuals have hypertension, it is critical to identify the underlying mechanism causing AAS. Progress in genetics and translational research may be useful in forecasting future risks of AAS.

The goal of using stabilising aortic wall medications, such as statins, beta-blockers or angiotensin receptor blockers, early in the course of a medical condition is to prevent the aneurysm from progressing [108]. Clinical investigations have demonstrated that statins have a preventive function in AD development and therapy. It has been established that the action of statins on AD seems unlikely to be dependent on the primary action of reducing cholesterol levels. In contrast, minichromosome maintenance proteins, a class of proteins that statins target, are increased in the tissues of the torn aorta wall and are crucial for controlling the cell cycle and mitosis in patients with AD. They have been used to stop the course of aortic illnesses, and there is growing evidence that they can reduce aortic enlargement and rupture [109]. It is still not readily apparent whether these or other medications reduce the risk of AAS in individuals without connective tissue disorders and with or without hypertension; this requires more investigation. The use of fluoroquinolones has been linked to an elevated risk of acute AD and aortic aneurysm, according to population-based research [110]. Furthermore, when these individuals are administered this type of antibiotic, they risk experiencing negative effects [107]. Thus, fluoroquinolones should be avoided in patients at risk for AAS if another treatment option is available [111].

## 5. Conclusions

Over the past 20 years, there have been significant changes in the diagnosis and management of AAS. AAS must be considered and identified as soon as possible in patients who present with acute chest or back pain and high blood pressure, due to high rates of mortality and morbidity. For AAS, CTA is the preferred diagnostic method. Improved surgical outcomes have led to a considerable decrease in mortality in patients with type A AAS. Because endovascular treatment has become more widely available, the use of medical treatment alone has declined for patients with type B AAS, but in-hospital mortality has not decreased considerably. Furthermore, there are still numerous unanswered questions about the diagnosis and management of AAS. To improve our understanding of the architectural and functional characteristics of the aortic wall, innovations and basic research should be prioritised. To find the disease early, precise biomarkers of AAS are required. It is advisable to expand access to centres for aortic surgery and institutional protocols specific to AAS. To determine the course of treatment for patients with less prevalent types of AAS, more research is required. The effectiveness of preventive therapies in the context of aortic problems needs to be tested by prospective multi-centre clinical trials and, more practically, mandatory registries.

## Figures and Tables

**Table 1:** Diagnostic tools for acute aortic syndromes: sensitivity and specificity.

Diagnostic Tool	Sensitivity	Specificity
Computed tomography	100	100
Magnetic resonance imaging	95.0–100	94.0–98.0
Transesophageal echocardiography	86.0–100	90.0–100
Transthoracic echocardiography	73.7–100	71.2–91.0
Intravascular ultrasound	100	100
D-dimer	51.7–100	32.8–89.2
Elastin degradation products	99.8	99.8
Matrix metalloproteinase 8/9	100	9.5
Smooth muscle myosin heavy chain	90	97
Soluble lectin-like oxidised low-density lipoprotein receptor 1	89.5	94.3

**Table 2:** Reported results of treatment for acute aortic syndromes (AASs).

	Medical	Open Surgical Procedure	TEVAR ^1^
Mortality range of AAS (%)	0–29	0–50	0–21

^1^ Thoracic endovascular aneurysm repair.

## Data Availability

The data supporting this study’s findings are available from the corresponding author upon request.
